# Association of Social Media Use and High-Risk Behaviors in Adolescents: Cross-Sectional Study

**DOI:** 10.2196/18043

**Published:** 2020-05-26

**Authors:** Teresa Vente, Mary Daley, Elizabeth Killmeyer, Laura K Grubb

**Affiliations:** 1 Ann & Robert H Lurie Children's Hospital of Chicago Northwestern University Feinberg School of Medicine Chicago, IL United States; 2 The Floating Hospital for Children at Tufts Medical Center Tufts University School of Medicine Boston, MA United States

**Keywords:** self-harm, social media, nonsuicidal self-injury, sexting

## Abstract

**Background:**

Previous studies have demonstrated the prevalence of social media use and identified the presence of high-risk behaviors among adolescents, including self-harm and sharing of sexually explicit messages.

**Objective:**

This study aimed to identify patterns in the amount of time spent on social media by adolescents who engage in high-risk behavior and the extent to which they use social media as a platform for sharing such behaviors.

**Methods:**

This was a descriptive cross-sectional study of 179 adolescents seen in a pediatric clinic at an urban medical center. We used an anonymous self-report survey to obtain demographic characteristics, rates of self-harm thoughts and behaviors, sharing of sexually explicit messages, and social media use as determined by total hours spent on social media per day and the number of applications used.

**Results:**

Most adolescents reported spending 3 to 5 hours on social media each day and using 3 or more social media applications. Almost 1 in 8 (22/179, 12.3%) adolescents self-reported having ever engaged in self-injury with a mean age of onset of 11.8 years. Over a quarter (49/179, 27.4%) of adolescents reported sharing sexually explicit messages. Relative risk of engaging in self-injury and or sharing sexually explicit messages increased with the use of 4 or more social media applications (1.66; CI 1.11-2.48).

**Conclusions:**

Results show a relationship between the number of social media applications used and increased rates of high-risk behaviors. We identified relevant risk factors that clinicians can use to screen for high-risk behavior and parents can monitor to encourage education about healthy online practices.

## Introduction

The degree to which social media has permeated our culture has led to growing concerns among parents and clinicians regarding how best to protect young people from vulnerabilities unique to the modern social media landscape [1-4]. Some of the most commonly used social media platforms originated when today’s adolescents were infants [5]. As a result, this type of technology has essentially been woven into their developmental experience, with unknown long-term impacts. Several studies have explored how internet and social media use influence adolescent behavior [6-9]. Social media platforms may be an important means to expose young people to the concept of self-harm and may provide a sense of normalcy or belonging via online communities of peers engaging in similar behaviors, thereby perpetuating these harmful behaviors [10-14]. Researchers have sought to identify trends in social media use and how they may relate to the concomitantly growing rates of self-injurious and suicidal behaviors, sharing of sexually explicit messages and photos, and other forms of potentially harmful activities [12,13,15].

Deliberate self-harm or nonsuicidal self-injury (NSSI) is the intentional and direct destruction or alteration of body tissue resulting in tissue damage without conscious suicidal intent [12,16]. This behavior is recognized in literature in both North America and Europe [16,17]. Experts estimate the prevalence of NSSI ranges from 4% to 35%, with higher rates found in psychiatric patients and significant variation depending on definition and measurements used [16,17]. Previous studies of adolescents who reported a history of self-injury found more than 50% had engaged in internet searches for self-injury material [8]. Qualitative interviews revealed while some adolescents reported discovering the concept of self-injury online and subsequently engaging in self-injurious behaviors, most used online platforms to support preexisting behaviors [13]. Existing evidence also shows online communities can perpetuate potentially dangerous behaviors through increased exposure and normalization [12-14]. A survey-based study of more than 700 participants demonstrated that youth who self-reported “unmet need for mental health support” had an adjusted relative risk ratio of 3.15 for spending more than 2 hours per day on social media and that adolescents who reported more than 2 hours of social media use per day also had an independent association with psychological distress including suicidal ideation [9,14,18]. Social media and online communities present opportunities for adolescents to seek validation and a sense of belonging around these behaviors. The ability to identify sources of potentially harmful content on the internet can be exceedingly difficult for parents and providers, highlighted by research specifically examining the presence of self-injury on the social media site Instagram, where users disguised posts with ambiguous hashtags making them difficult to detect and content warnings were often not reliable [12].

Sexting is a term used to refer to the act of sending or receiving sexually explicit or sexually suggestive photos, videos, or messages. Prior studies estimated the prevalence of adolescents sharing sexually explicit images and/or videos via text messaging or social media ranges from 5% to 7%, with rates of sharing sexually explicit messages (without images) as high as 22% [19,20]. Studies found the most commonly reported reasons for sexting among teenagers include responding to a request from a partner or seeking to attract interest or increase popularity [21]. The same studies showed girls are more likely to be pressured, coerced, or blackmailed into sexting, particularly by male peers [21]. Sexting has been shown to be associated with both sexual activity and substance use [22]. Researchers have not fully studied relationships between sexting and self-injurious behaviors. A 2014 research review suggested that, despite common perceptions, rates of cyberbullying, contact with strangers, and sharing of sexually explicit content did not appear to rise with increased access to the internet or mobile technology, and these behaviors were more likely attributable to psychosocial risk factors. Authors have postulated that mass media campaigns for widespread social awareness and safety initiatives may mitigate risk for engaging in such behaviors [23].

A more comprehensive understanding of current social media practices among adolescents is essential to implement efforts to identify adolescents in crisis, educate parents and adolescents about how to respond to high-risk behaviors, and protect vulnerable adolescents both from self-destructive behaviors and victimization. Our study aimed to identify patterns in time spent on social media by adolescents who engage in NSSI and/or sexually explicit sharing on social media compared with those who do not and the extent to which adolescents use social media as a platform for sharing and thereby possibly perpetuating such behaviors.

## Methods

### Study Design

We recruited study participants from the outpatient general pediatric and adolescent medicine clinic at an academic urban medical center from August 2016 to May 2018. The institutional review board approved this study and granted a waiver for parental consent of participants aged younger than 18 years. We defined adolescents according to those outlined by the age limitations of the American Academy of Pediatrics (AAP) [24,25]. Adolescents were eligible to participate if they were attending clinic for any visit, were between the ages of 12 and 21 years, understood spoken and written English, and had no documented intellectual disability. Participants 18 years or older provided verbal and written informed consent prior to participation. For participants aged younger than 18 years, we obtained verbal and written assent to participate. We administered a survey to eligible participants onsite in clinic and collected them at the end of their appointment. Self-reported survey responses were anonymous, and we coded surveys with deidentified information. Researchers reviewed surveys upon collection for any concern of actively occurring self-injurious behavior and discussed any concerns with the participant’s provider to determine need for additional safety intervention. Participants could withdraw from the study at any time.

### Measures

Authors designed a 40-question survey tool that collected basic demographic information (age, gender, ethnicity), grade level in school, history of being bullied, NSSI thoughts and behaviors, behaviors related to sharing of sexually explicit messages (sexting), and social media use. We based questions related to NSSI thoughts and behaviors on validated survey tools including the Self-Harm Inventory and the Self-Injurious Thoughts and Behaviors Interview [26,27]. These questions recorded responses related to frequency, duration, method, and communication of NSSI thoughts and behaviors. Survey questions also recorded responses to frequency, duration, and type of sexually explicit messages shared with questions based on the proposed Sexting Behaviors Scale [28]. This proposed survey tool was used as a guide as validated measures of sexting behaviors have not been well developed or studied [29]. Questions regarding social media, recorded total time spent on social media per day, and the number and type of social media applications used were also included. We also surveyed adolescents on how they use social media (messaging/texts, photos, videos, meeting people, or other). All survey responses for high-risk behaviors were self-reported.

### Statistical Analysis

Statistical analysis was performed using SPSS Statistics version 22 (IBM Corp). We used descriptive statistics to summarize numerical survey data. We used chi-square tests to assess for associations between categorical variables and computed the association of social media use with high-risk behaviors as relative risks and reported confidence intervals as 95%. Due to missing data, there is some variability in denominators across variables.

## Results

### Participant Demographics

A total of 179 adolescents completed anonymous surveys. Most survey respondents were female (120/179, 67.0%). The mean age of respondents was 16.8 (SD 2.5) years. The sample of adolescents was ethnically diverse and included white, black, Hispanic, and Asian adolescents, with Asian adolescents making up the largest percentage of respondents (53/179, 29.6%). The highest percentage of respondents reported parents as married (76/179, 42.4%). Table 1 provides additional demographic data.

A total of 41.3% (74/179) of adolescents reported being bullied, with verbal threats, taunting, or teasing as the most common type of bullying (53/179, 29.6%). A statistically significant difference was found in the number of bullied male respondents versus the number of bullied female respondents (24.0% [14/58] vs 49.0% [59/118]; *P*=.001). There was no statistically significant difference in being bullied reported across race.

**Table 1:** Participant demographics (n=179).

Variable		Value
**Gender, n (%)**		
	Male	58 (32.4)
	Female	120 (67.0)
	Other	1 (0.6)
Age in years, mean (SD)		16.8 (2.2)
**Grade level, n (%)**		
	Sixth	4 (2.2)
	Seventh	4 (2.2)
	Eighth	17 (9.5)
	Ninth	14 (7.8)
	Tenth	16 (8.9)
	Eleventh	28 (15.6)
	Twelfth	21 (11.7)
	College	33 (18.4)
	Missing	42 (23.5)
**Average grades, n (%)**		
	Mostly As	46 (25.7)
	Mostly Bs	90 (50.3)
	Mostly Cs	25 (14.0)
	Mostly Ds	0 (0)
	Missing	18 (10.0)
**Race/ethnicity, n (%)**		
	White, non-Hispanic	36 (20.1)
	White, Hispanic	16 (8.9)
	Black, Hispanic	13 (7.3)
	Black, non-Hispanic	41 (22.9)
	American Indian/Alaska Native	0 (0)
	Asian	53 (29.6)
	Other	15 (8.4)
	Missing	5 (2.8)
**Being bullied, n (%)**		
	Yes	74 (41.3)
	No	103 (57.5)
	Missing	2 (1.1)
**Parent marital status, n (%)**		
	Married	76 (42.5)
	Single	43 (24..0)
	Separated	24 (13.4)
	Divorced	24 (13.4)
	Widowed	3 (1.7)
	Other	5 (2.8)
	Missing	4 (2.2)

### Social Media Use

A total of 93.8% (168/179) of adolescent respondents reported at least 1 hour of social media use per day, with 3 to 5 hours of social media use per day being the most common (63/179, 35.2%).

Most adolescents reported mixed use of social media with 75.4% (135/179) using social media for messaging, texting, or online chatting. Of those adolescents who reported using social media, nearly 40% (71/179, 39.7%) used 4 or more social media platforms. There was no difference in social media use by race or gender.

### Self-Harm (Nonsuicidal Self-Injury)

One in five adolescents endorsed a history of having thoughts of self-harm (35/179, 19.6%). The mean age of first thoughts of self-harm was 11.8 (SD 2.5) years. Of those adolescents who reported having thoughts of self-harm, more than half (20/35, 57%) reported those thoughts occurring a few times per year, and 69% (24/35) had shared those thoughts with their friends. More than 40% (78/179, 43.6%) of adolescents reported knowing their friends had self-harm thoughts. Of the adolescents who reported thoughts of self-harm, 74% (26/35) reported being bullied compared to 33.3% (47/141) of adolescents who reported no thoughts of self-harm (*P*<.001). Females reported more self-harm thoughts than males (82% [28/34] vs 18% [6/34]; *P*=.01). Asian adolescents reported more self-harm thoughts than adolescents of any other racial group (12/31, 39%, *P*=.01).

Nearly 1 in 8 (22/179, 12.3%) adolescents endorsed a history of engaging in self-injurious behaviors and reported cutting as the most common behavior (17/22, 77%). The mean age of first engaging in self-harm was 11.8 (SD 4.6) years. Of those adolescents who reported engaging in self harm, 41% (9/22) engaged in self-harm a few times per year. Almost one-third (56/179, 31.2%) of adolescents reported knowing their friends engaged in self-injurious behaviors. Of those adolescents who communicated with their friends about engaging in self-harm, more than a quarter (10/34, 29%) communicated those thoughts via social media. Of the adolescents who reported engaging in self-injurious behaviors, 68% (15/22) reported being bullied, compared with 36.7% (47/128) of adolescents with no self-injurious behaviors (*P*=.06). Females reported more self-injurious behaviors than males (77% [17/22] vs 18% [4/22]; *P*=.001). Other race adolescents reported more self-harm compared adolescents of any other racial group (10/22, 45%; *P*<.001).

### Sexting

Two-thirds (120/179, 67.0%) of adolescents reported texting with friends more than once per day. Over a quarter (49/179, 27.4%) of adolescents reported that they shared sexually explicit messages with others, with texting as the most common form of communication (41/49, 84%). Adolescents shared sexually explicit messages with multiple people, with boyfriends and girlfriends as the most common (34/49, 69%). Of those adolescents who shared sexually explicit messages, most responded they did it when someone asked for them (12/49, 25%). Almost one-third (57/179, 31.8%) of adolescents knew of someone who had a bad experience from sharing sexually explicit messages. There was no difference in sexting across race or gender. Tables 2 and 3 provide a summary of self-harm and sexting behavior reported in adolescents.

**Table 2:** Summary of self-harm behaviors reported by adolescents (n=22).

Characteristic		Value, n (%)
**Self-harm subtype**		
	Cutting	17 (77)
	Burning	3 (14)
	Scratching	10 (46)
	Hitting	14 (64)
	Skin picking	7 (32)
	Other	0 (0)
**Frequency**		
	Every day	0 (0)
	A few times per week	5 (23)
	A few times per month	6 (27)
	A few times per year	9 (41)

**Table 3:** Summary of sexting behaviors reported by adolescents (n=49).

Characteristic		Value, n (%)
**Sexting subtype**		
	Text	41 (84)
	Photo of self	25 (51)
	Photo of someone else	29 (59)
	Video of self	7 (14)
	Video of someone else	8 (16)
**Recipient**		
	Classmates	11 (22)
	Boyfriend/girlfriend	34 (69)
	Other friends/neighbors	10 (20)
	Online friends	4 (8)
**Reason**		
	Someone asked	12 (25)
	Without being asking	6 (12)
	In return for photos	11 (22)
	Peer pressure	2 (4)
	Other	19 (39)

### Risk of Self-Harm (Nonsuicidal Self-Injury)

Table 4 shows the relative risks for thoughts of self-injury, self-injurious behaviors, sexting, or any of those behaviors compared with the amount of social media use, including hours per day and number of applications used. The relative risk of any high-risk behavior (self-harm, sexting, or both) was 1.66 in adolescents who used 4 or more social media applications at a time (95% CI 1.11-2.48). Relative risk of high-risk behaviors was greater than 1.00 for social media use of more than 5 hours per day, but these findings were not statistically significant.

**Table 4:** Risk of self-harm/risk-taking behavior with social media use.

Social media use and self-harm/risk-taking behavior		Relative risk (95% CI)
**Any social media use**		
	Thoughts of self-harm	0.48 (0.16-1.46)
	Self-harm behaviors	0.45 (0.09-2.32)
	Sexting	0.69 (0.63-0.77)
	Self-harm, sexting, or both	1.22 (0.24-6.13)
**Use of 4 or more social media applications**		
	Thoughts of self-harm	1.02 (0.55-1.87)
	Self-harm behaviors	1.33 (0.62-2.88)
	Sexting	1.96 (1.00-3.87)
	Self-harm, sexting, or both	1.66 (1.11-2.48)
**>5 hours of social media use per day**		
	Thoughts of self-harm	1.11 (0.59-2.06)
	Self-harm behaviors	1.31 (0.60-2.84)
	Sexting	1.57 (0.99-2.50)
	Self-harm, sexting, or both	1.32 (0.8-1.98)

## Discussion

### Principal Findings

Our results suggest a positive association between the number of social media platforms used and increased rates of high-risk behaviors in adolescents, including NSSI and sharing of sexually explicit content. This was evidenced by the relative risk of 1.66 (95% CI 1.11-2.48) for any high-risk behavior (self-harm, sexting, or both) in adolescents who reported use of 4 or more social media applications. Although not statistically significant, our data implies increased rates of sharing of sexually explicit messages in respondents who reported more than 5 hours of social media use per day (relative risk 1.57; 95% CI 0.99-2.495). These findings are particularly relevant as adolescents overall reported a large amount of daily social media use, with nearly all of those surveyed meeting the criteria for heavy use [30].

The amount of social media use that adolescents self-reported in this study is not surprising given the findings of the latest report issued by the Pew Research Center in 2018 where 45% of teens reported being online almost constantly, an increase of more than 20% from 2014-2015 [31]. Diverse use and engagement in multiple online social media platforms as reported by the adolescents in our study are also consistent with data from the same report by the Pew Research Center in which teens reported rates of use across multiple platforms [31]. With the advent of social media applications directed for text-based communication, adolescents also have the capacity to exchange greater numbers of texts and messages [32].

Increased screen time and digital media use have previously been associated with other health risks in children and adolescents, including obesity and cardiovascular risk as well as decreased sleep and poorer sleep quality [32,33]. Greater attention has also been brought to the mental health risks of social media use including higher rates of depression (also known as Facebook depression) and anxiety [34,35]. High-risk behaviors including rates of alcohol consumption have been associated with increased social media use as well [36]. It is therefore not surprising that engaging in additional high-risk behaviors such as those examined in this study would have a similar association with increased social media exposure.

While our study surveyed patients ages 12 to 21 years, the results indicate that the average age at which young people first experienced thoughts of self-harm and the average age at which they report first engaging in self-injurious behaviors was 11.8 years. This is consistent with existing literature which generally reports age at onset of self-injurious behaviors to range from 11 to 15 years and suggests a need for further study among younger patients [37,38]. Also similar to prior studies, we found youth identified cutting as the most common method of self-harm and females were substantially more likely than males to endorse thoughts of self-harm and report a history of engaging in NSSI [39].

Adolescents in our study reported awareness of friends engaging in self-harm, and social media was identified as method by which adolescents self-reported sharing thoughts and actions of self-harm with others. Some social media platforms have taken steps to limit the sharing of explicit photos or messages of self-harm [1,40]. Adolescents who are aware of and exposed to posts about self-harm on social media may experience changes in their perception or attitude toward self-harm behavior including normalization [41]. Given the speed at which new social media platforms are being developed, a significant challenge remains for developers to be able to moderate content and promote safety for all users.

In addition to identifying these specific risk factors, our study generates concern for the growing rates of these high-risk behaviors seen in adolescents of all ages, genders, and backgrounds—not just adolescents referred for psychiatric evaluation or treatment [42]. Our results found the prevalence of non-suicidal self-injurious behaviors consistent with existing evidence but revealed higher rates of sharing of sexually explicit messages than previously estimated [12,17].

There is concern that adolescents may not fully appreciate the consequences of sharing sexually explicit content through texting or social media and are particularly uneducated regarding issues related to privacy [32]. There are discrepancies in what adolescents believe to be public vs private content [43]. Teens may also be taking advantage of hidden apps or storage apps in an effort to shield online behavior from parents [43]. This may limit the knowledge parents have about adolescent behavior online, which in turn could significantly limit the opportunity for discussion between parents and teens regarding safe internet practices. The AAP has promoted raising awareness of healthy online behaviors and offers a guide to parents on how to create a family media use plan [44].

Additional studies to expand upon our findings and further explore how specific patterns of social media use relate to increased risk of high-risk behaviors will help to delineate more concrete thresholds (ie, duration daily of use, number of platforms used) that can be used for counseling and screening purposes. Areas of future study may include evaluation of the relationship between social media use and psychiatric comorbidities, with additional focus on preadolescent behavior, as well as identification of protective factors that might inform clinicians and parents how best to foster healthy use of social media in children and adolescents. Due to previously identified risk, efforts to screen for social media and internet use have been recommended by American College of Obstetrics and Gynecology as well as the AAP for adolescents during clinical visits [45,46].

### Limitations

Limitations of our study include the small sample size based on a nonrandom sample and lack of generalizability due to our sample population being taken from a single urban medical center. A larger sample size could potentially show a stronger association between the extent of social media use and incidence of high-risk behaviors. While we had a relatively racially heterogeneous sample, our results may not necessarily be generalizable to the population at large as our study was conducted in an urban medical center and our sample consisted of a majority of females and higher proportion of Asian adolescents. In addition, respondents were limited to those able to understand written and spoken English, which likely introduced further bias to our results. Although participants were able to self-report behaviors anonymously, due to the sensitive nature of some survey questions, some reporting bias is expected. Lastly, we did not assess for comorbidities. Further studies may assess whether adolescents with psychiatric comorbidities such as anxiety or depression are more likely to engage in more extensive social media use and whether there are discrepancies in risk when comparing adolescents with and without psychiatric comorbidities.

### Conclusion

Social media access is ubiquitous among adolescents. With high rates of self-reported use, there is also concern for engagement in other high-risk behaviors. This study demonstrates the potential for increased risk of NSSI and exchange of sexually explicit messages with higher levels of social media use. Sharing of self-harm practices on social media or using social media to share sexually explicit content as reported by adolescents in this study may essentially normalize these high-risk behaviors. Although there are notable limitations to this study, it highlights the importance of screening for social media use, including duration of daily use and number of applications used, so that clinicians and parents may have an opportunity to address concerns and provide guidance and education on safe internet use for adolescents and their families.

## Abbreviations

AAP: American Academy of Pediatrics
NSSI: nonsuicidal self-injury
